# Association of Two Polymorphisms in CCL2 With Parkinson's Disease: A Case-Control Study

**DOI:** 10.3389/fneur.2019.00035

**Published:** 2019-01-29

**Authors:** Ruinan Shen, Suzhen Lin, Lu He, Xue Zhu, Zhekun Zhou, Shengdi Chen, Ying Wang, Jianqing Ding

**Affiliations:** ^1^Institute of Neurology, Ruijin Hospital, Shanghai Jiao Tong University School of Medicine, Shanghai, China; ^2^Shanghai Jiaotong University School of Medicine, Shanghai, China

**Keywords:** CCL2, polymorphism, Parkinson's disease, Chinese population, risk factor

## Abstract

**Background:** Parkinson's disease (PD) is the most common neurodegenerative movement disorder that is known to be related to neuro-inflammation. Chemokines participate in this process usually through upregulation of expression levels, which are closely related to the polymorphisms in their genes. Recent studies have further revealed the association between these polymorphisms and the risk of PD in multiple populations, but not the Chinese Han population.

**Methods:**The promoter region of CCL2 was sequenced in 411 PD patients and 422 gender-age matched control from a Chinese Han population using PCR-RFLP method. Their genotype frequencies were analyzed statistically. Dual-luciferase reporter assays were conducted in neuroblastoma cells to assess the promoter transcriptional activity of the rs1024611 variants (T>C) and the GRCh38.p12chr17:34252593 G>C alleles in CCL2.

**Results:**We found that the frequency of the CCL2 genotype of rs1024611 was significantly different between the PD and control groups (*p* = 0.021), while the C allele was associated with a significantly increased risk in the PD group (*p* = 0.004). Moreover, C allele of this newly identified alteration in CCL2 (GRCh38.p12chr17:34252593 G>C) was also found to be associated with an increased risk of PD (P genotype = 0.006, P allele = 0.006). Dual-luciferase reporter assay results indicated that rs1024611 C allele and GRCh38.p12chr17:.34252593 C allele increased the transcriptional activity of the CCL2 promoter.

**Conclusions:** We, for the first time, report a risk polymorphism (rs1024611) and a new locus (GRCh38.p12chr17:.34252593 G>C) on CCL2, both of which are suggested as risk factors for PD in a Chinese Han population.

## Introduction

Parkinson's disease (PD), a complex neurodegenerative disorder characterized by bradykinesia, resting tremor, muscular rigidity, and postural instability, affects at least 1% of the population over 60 years old worldwide (1). These clinical manifestations are the consequences of dopaminergic neuron loss in the substantia nigra pars compacta (SNpc) of the midbrain (2). Although the cause of the neuron loss is unclear, accumulating lines of evidence suggest that neuro-inflammation is an important participant in the process of neurodegeneration (3), including the abnormal activation of microglia, the presence of cytotoxic T-lymphocytes in the SNpc adjacent to blood vessels and dopaminergic neurons, and increased concentrations of multiple chemokines in the striatum, serum, or cerebrospinal fluid (4).

Chemokines are a large family of cytokines with homologs structures, and they play crucial roles including attracting cytotoxic T-lymphocytes and activating microglia in neuro-inflammation (5). Chemokines and their receptors, such as MCP-1/CCR2, fractalkine/CX3CR1, SDF-1α/CXCR4, MIP-1α/CCR5, IP-10/CXCR3, IL-8/CXCR1, CXCR2, and RANTES/CCR1, CCR3, CCR5 are widely involved in neuroinflammation, they are produced by activated astrocytes to induce immune cell migration and induce inflammatory cascade (6). Among them, monocyte chemoattractant protein-1 (MCP1 or CCL2) is widely reported upregulated in PD (7, 8). CCL2 is a potent chemotactic factor for monocytes (9). Altered expression level of CCL2 might lead to microglia over-activation and/or induce neuron damage, then finally lead to neuroinflammation (10), which will further evolve into neurodegenerative diseases including PD, Alzheimer's disease, and amyotrophic lateral sclerosis (11). Reale et al. reported that the increased serum level of CCL2 in PD patients might be related to the pathogenesis of neurodegeneration (8). Although there is no direct evidence proving their upregulation in brain tissue, elevated expression level of CCL2 in peripheral monocytes is detected (7). Microglia are the resident macrophages of the central nervous system which share a lot of features with peripheral, so the change of chemokines profile in monocytes might represent what happen in microglia to a certain degree. Since the protein expression level is closely related to gene transcription, certain single nucleotide polymorphisms (SNPs) in the transcription regulatory region of CCL2 might affect their expression level in PD patients. Rovin et al. reported that the C allele of rs1024611 in CCL2, located 1.8–2.7 kb upstream of the transcriptional start site of CCL2 (12), increased the risk of early-onset PD. They found PD patients who carry one or more C allele of rs1024611 had earlier onset age than those who carry allele T (55.2 year-old for heterozygous CT or 54.1 year-old for homozygous CC for vs. 61.9 year-old for homozygous TT) (13). From these studies it is clear that SNPs located in the promoter region of CCL2 might lead to altered expression levels of the protein (14).

Although these SNPs have been studied in several distinct ethnic populations, any similar case control study has not yet been performed in a Chinese population. Given that the genetic factors of diseases might be ethnicity specific, we recruited 411 PD patients and 422 controls from a Chinese Han population. We focused on the transcription factor binding site regions in CCL2 because polymorphism in this region might affect their promoter activity and previous studies have shown the relationship between these changes and PD.

## Materials and Methods

### Subjects

All subjects recruited had the same ethnic background (Table 1). The patient group was composed of 411 Chinese PD patients from mainland China (205 males and 206 females; mean age 65.32 ± 7.22 years). Patients were enrolled from the Movement Disorders Center Clinic at Department of Neurology, Ruijin Hospital affiliated with Shanghai Jiaotong University School of Medicine. The PD diagnosis was made in accordance with the UK Parkinson's Disease Society Brain Bank's criteria (15). Patients with clinical features of Parkinson's-plus syndrome such as extensor plantar reflexes, ophthalmoplegia, early dementia, or early autonomic failure were excluded. The modified Hoehn and Yahr scale (H-Y) was rated in the OFF state of each patient. PD patients were divided into Tremor Dominant (TD) (*n* = 72), Akinetic/Rigid (AR) (*n* = 91), and Mixed (MX) (*n* = 248) subtypes with the criteria used in previous studies (16) based on agreement between two clinicians who specialized in movement disorders. A total of 422 gender-age matched subjects without neurological disorders were established as a control group (200 males and 222 females; mean age 65.49 ± 7.34 years). Basic medical information, including on hypertension (HTN) and type 2 diabetes mellitus (D2M), were collected. The incidence of HTN in our control population, did not differ from that found in the Chinese population, which is about 43% (17). We received approval from the Ethics Committee of Ruijin Hospital affiliated with Shanghai Jiao Tong University School of Medicine. Informed consent for participation in the study was obtained from all subjects.

**Table 1:** Characteristics of the study population.

		**Patient**	**Control**	***P*-value**
Total sample (N)		411	422	–
Male (N)		205	200	0.473
Female (N)		206	222	
Age (Mean ± SD)		65.32 ± 7.22	65.49 ± 7.34	0.746
50~59(N)		96	103	
~69(N)		196	196	0.979
~79(N)		109	104	
~89(N)		14	14	
Age of onset (Mean ± SD)		61.07 ± 7.03	N/A	
Classification	TD	72	N/A	
	AR	91		
	Mixed	248		
H-Y Scale		2.428 ± 0.772	N/A	
Time since PD diagnosis (month)		56.84 ± 46.58	N/A	
Hypertension		55	170	<0.001
Diabetes		13	45	<0.001
Smoke		109	100	0.472
Alcohol		60	77	0.132

### Genetic Analysis

Genomic DNA was extracted from peripheral blood through the standardized phenol/chloroform extraction method. The *CCL2(rs1024611)* polymorphisms was determined by the polymerase chain reaction-restriction fragment length polymorphism technique (PCR-RFLP) (Table 2). After amplification, the products were purified and sequenced (BigDye terminator v3.1) using an ABI 3730XL DNA sequencer (Applied Biosystems).

**Table 2:** PCR primers.

**Gene**	**Primer sets**	**PCR product size (bp)**	**Tm (^**°**^C)**
MCP-1	For:5′-TTTCCCTTGTGTGTCCCCAAG-3′	976	^*^TD
(rs1024611)	Rev:5′-CTGCTTTGCTTGTGCCTCTT-3′		
MCP1 vectors	For: 5′- CGGGGTACCTTTCCCTTGTGTGTCCCCAAG-3′ Rev: 5′- CCGCTCGAGCTGCTTTGCTTGTGCCTCTT-3′	985	^*^TD

* *The touch down (TD) of PCR means a programmed temperature reduction from 68 to 53°C in 10 cycles (1.5°C/cycle), continuing with 20 cycles of 53°C during annealing stage*.

### Construction of Luciferase Reporter Gene Vectors and Luciferase Reporter Assays

The CCL2 promoter region was amplified from the genomic DNA of PD patients using primers in Table 2. The PCR products were digested and then cloned into pGL3-basic luciferase plasmids (Promega, Beijing, China). The reporter plasmids were named p-T (Wild Type) or p-C (rs1024611) and n-G (Wild Type) or n-C (GRCh38.p12chr17:34252593 G>C), respectively.

Human neuroblastoma cells (SH-SY5Y) were cultured in DMEM with 10% FBS (GIBCO/Invitrogen, Shanghai, China). SH-SY5Y cells were plated into 24-well culture plates 24 h, and the confluency of the cells were about 80% prior to transfection. p-C, p-T, n-G, n-C, or pGL3-basic empty plasmid (as a negative control) of 490 ng was transfected into SH-SY5Y cells, respectively, using Lipofectamine 3000 (Invitrogen, Shanghai, China). Renilla pRL-SV40 plasmid (Promega, Beijing, China) of 10 ng was co-transfected as a normalizing control. Transcriptional activity was determined using the Dual-Luciferase Reporter Assay System (Promega, Beijing, China) on a Synergy™ H4 Hybrid Microplate Reader (Biotek, Shanghai, China). For each sample, the readings were taken in duplicate. Transcriptional activity was reported as the relative luciferase activity. This was the ratio of firefly luciferase activity over Renilla luciferase activity.

### Meta-Analysis

In order to have a better understanding of the relationship between these SNPs and PD, we performed a Meta-analysis. We designed a search strategy using 3 English language databases including PubMed, Embase, and Cochrane Library. The following principal search terms were used: “MPC1” or “CCL2” or “Monocyte chemoattractant protein-1” or “rs1024611” or “MCP-1−2518A/G” and “Parkinson's disease” or “PD” and “SNP” or “single nucleotide polymorphism.” We looked for additional studies in reference lists of included articles too. The results were crosschecked to eliminate duplicates. Articles were retrieved through April 2018. The following studies were included in analysis regardless of race, sex; patients needed to accept sequencing of *CCL2* gene promoter polymorphism (sequence methods are not limited); and studies published in English. Studies with incomplete or incorrect information were excluded from the analysis. Meta-analysis was conducted with RevMan 5.3. The data was pooled and analyzed for Odds ratio (OR) with 95% confidence interval (CI). Assessment of heterogeneity was done by *I*-squared (*I*^2^) statistics. A fixed-effects model (Inverse Variant method) was initially conducted. If significant heterogeneity was found among trials (*I*^2^ > 50%), a random-effects model (Mental-Haenszel method) was used.

### Statistical Analysis

All statistical analyses were performed using SPSS software. For analyzing individuals' demographic statistics, independent the *t*-test or one-way ANOVA was used for continuous variables (age, age at onset). The Mann-Whitney U test was used for discrete variables (Hoehn and Yahr stage), and the chi-square test or Fisher's Exact test was used for nominal data (gender, HTN, D2M, never-smokers, never-drinkers). The chi-square test or Fisher's exact test was used to assess the deviation of alleles in Hardy-Weinberg equilibrium and to evaluate the differences in genotype and allele distributions between groups. Each genotype was estimated by logistic regression analysis presuming additive, dominant, and recessive modes of inheritance under correcting for confounders (HTN, D2M). A two-tailed *P* < 0.05 was considered statistically significant. For stratified analysis, a two-tailed *P* < 0.025 was considered statistically significant. For multiple statistical tests, the Bonferroni method was applied to correct the alpha level and *P*-values accordingly. For luciferase assay data, *t*-test were carried out between WT and alteration for each SNP.

## Results

### Demographic and Clinical Characteristics of the Participants

The patients and controls recruited for this study were well-matched by gender and age. The general data on the 833 participants are displayed in Table 1. Compared to the controls, PD patients had significantly lower rates of hypertension and D2M. The smoking and drinking histories of PD patients were equal to those of the control group, which was consistent with several other studies (18, 19).

### Single-Point Mutation Association Analysis of the Entire Population of PD Patients and Controls

**Figure 1:**
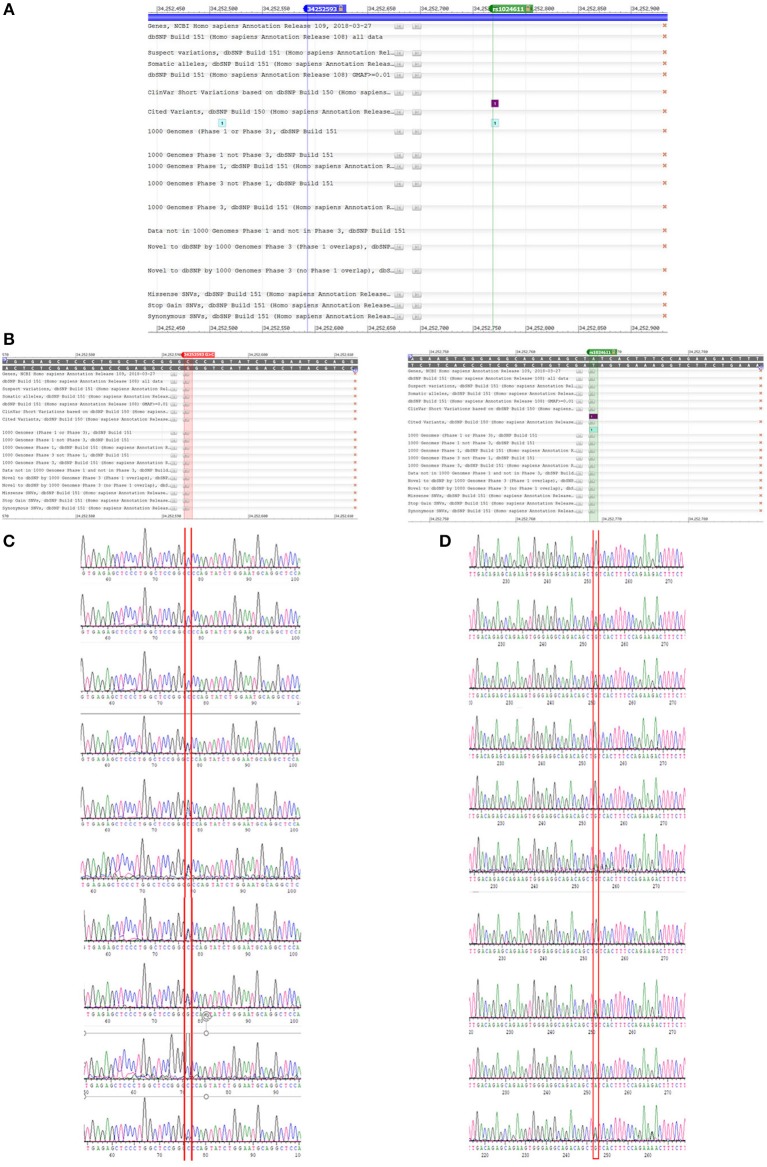
A newly detected mutation GRCh38.p12chr17:.34252593 G>C in CCL2. **(A,B)** We amplified promoter region of CCL2 and sequenced the anti-sense strand of it. Then we compared our sequence data with data on NCBI dbSNP database, and found rs1024611 and GRCh38.p12chr17:.34252593 G>. Unlike rs1024611 green line in **(A)** and green box in **(B)**, GRCh38.p12chr17:.34252593 G>C blue line in figure **(A)** and red box in figure **(B)** has no annotation in the database. **(C)** Shown are three control (homozygous GG) and seven heterozygous (GC) PD patients for the GRCh38 p12chr17:.34252593 allele. Base pair in red square is GRCh38.p12chr17:.34252593. Double peak with ratio >1:3 will be recognized as heterozygous. **(D)** Sequence of rs1024611 of the same 10 subjects above.

Among the three gene segments we amplified and sequenced, two polymorphisms were found, rs1024611 is not significantly deviated from Hardy-Weinberg equilibrium (*p* > 0.05). The C allele of rs1024611 located in the hypothetical promoter region of CCL2 was significantly higher in PD patients (64.66%) than in the controls (57.50%), suggesting its association with an increased risk of PD (*p* = 0.004). In addition, a point mutation, GRCh38.p12chr17:34252593 G>C in CCL2 was identified for the first time. This single point mutation located 176 bp upstream of rs1024611 (Figure 1). The original allele is G and seven PD patients (1.70%) were heterozygotes who carried the mutant allele C. None of the controls carried allele C, also indicating a pathogenic effect of this mutation (*p* = 0.006) (Table 3).

**Table 3:** Association between genotype and allele of polymorphisms and sporadic Parkinson's disease.

**Gene**	**SNP**	MAF^*^		**Group**	HWE^*^	**Genotype(N)**			***P*-value**	**Allele(N)**		OR^*^(95% CI^*^)^**1−β**^
CCL2	rs1024611	EAS EUR	0.547 0.316			TT	CT	CC	**0.021**	T	C	0.746
		AFR AMR	0.228 0.486	PD		58	166	175		282	516	(0.610–0.911)
		SAS	0.321	Control	0.384	80	186	143		346	472	***P*** **=** **0.004**^**0.5329**^
CCL2	GRCh38.p 12chr17:.3 4252593	N.A.				GG	CG	CC		G	C	1.009
				PD		392	7	0	**0.006**	791	7	(1.002–1.012)
				Control	N/A	422	0	0		844	0	***P*** **=** **0.006**^**0.7631**^

* *HWE, Hardy-Weinburg equilibrium; MAF, Minor Allele Frequency, represent frequency of alternated allele in different populations, including; EAS, East Asian; EUR, European; AFR, African; AMR, Ad Mixed American; SAS, South Asian; (data from Allele Frequency Database) OR, odds ratio; CI, Confidence Interval. Bold indicates statistical significant values*.

### Single-Point Mutation Association Analysis of a PD Genetic Model

In terms of rs1024611 in CCL2, the alternative T allele was found to be significantly lower in the PD group than in control group (*p* = 0.004, OR = 0.746 [0.610–0.911]), suggesting its protective role in PD. After removing the confounding factors of gender and age with binary logistic regression, the analysis of the possible genotype model revealed that the homozygous model (*p* = 0.011, OR = 0.529 [0.396–0.887]), dominant model (*p* = 0.010, OR = 0.688 [0.518–0.914]), and additive model (*p* = 0.006, OR = 0.762 [0.627–0.925]) were meaningful. In addition, in this study, a new alteration in CCL2 was found, GRCh38.p12chr17: 34252593 G>C. In the PD group, 1.70% carried a C alteration different from the ancestral homozygous GG, while all of the controls were homozygous GG (*p* = 0.016). This indicated that the dominant model (*p* = 0.006, OR = 1.018 [1.005–1.031]) was a risk genotype model for PD patients (Table 4).

**Table 4:** Genetic model of polymorphism.

**Gene**		**CCL2**	
**SNP**		**rs1024611**	**34252593 G>C**
Homozygous	OR	0.592	N/A
	CI (95%)	0.396–0.887	N/A
	*P*-value	**0.011**	N/A
Dominant model	OR	0.688	1.018
	CI (95%)	0.518–0.914	1.005–1.031
	*P*-value	**0.01**	**0.006**
Recessive model	OR	0.699	N/A
	CI (95%)	0.483–1.013	N/A
	*P*-value	0.058	N/A
Additive model	OR	0.762	N/A
	CI (95%)	0.627–0.925	N/A
	*P*-value	**0.006**	N/A

*Bold indicates statistical significant values*.

### Single-Point Association Analysis of Subgroups Stratified by Different Factors

One of the clinical characteristics of PD is that its incidence among males is higher than that among females. To determine if the distributions of these SNPs were different between males and females, we stratified the subjects by their gender. The risk allele C of rs1024611 in CCL2 was found to be significantly higher in male patients (Table 5).

**Table 5:** Stratification analysis of the four SNPs.

**SNPs**		**rs1024611**	**GRCh38.p12chr17:.34252593 G>C**
Gender	Male	**0.013**	0.030
	Female	0.144	0.224
Classification		0.025	**0.005**
(AR/TD/MX)			
HTN	Without	**0.003**	0.043
D2M	Without	**0.003**	**0.002**

*All data shown are p-value. Bold indicates statistical significant values*.

PD patients were divided into three subgroups according to their clinical characteristics, and the associations of the SNPs in these subgroups of PD were studied. Only the newly detected alteration in CCL2, GRCh38.p12chr17:.34252593 G>C, was found to be significantly higher in the AR group (*p* = 0.005) after Bonferroni adjustment, while other subgroups lacked statistic differences (Table 5).

Some of the subjects in our study had a medical history of either HTN (27.101%) or D2M (6.963%). Since HTN and D2M are highly relevant to chronic inflammation, in HTN and D2M patients, upregulated cytokines and activated immune cells were found, and lead to further vascular inflammation and chronic inflammation in other organs (20). To eliminate any possible effects of HTN and D2M, we further excluded the HTN or D2M patients in the subgroups. We found that both rs1024611T>C and the new variant GRCh38.p12chr17:.34252593 G>C were significantly higher in the PD group in subjects without HNT (*p* = 0.003 and *p* = 0.043, respectively) or D2M (*p* = 0.003 or *p* = 0.002, respectively) (Table 5).

### Effects of rs1024611 and GRCh38.p12chr17: 34252593 G>C on CCL2 Promoter Transcriptional Activity

**Figure 2:**
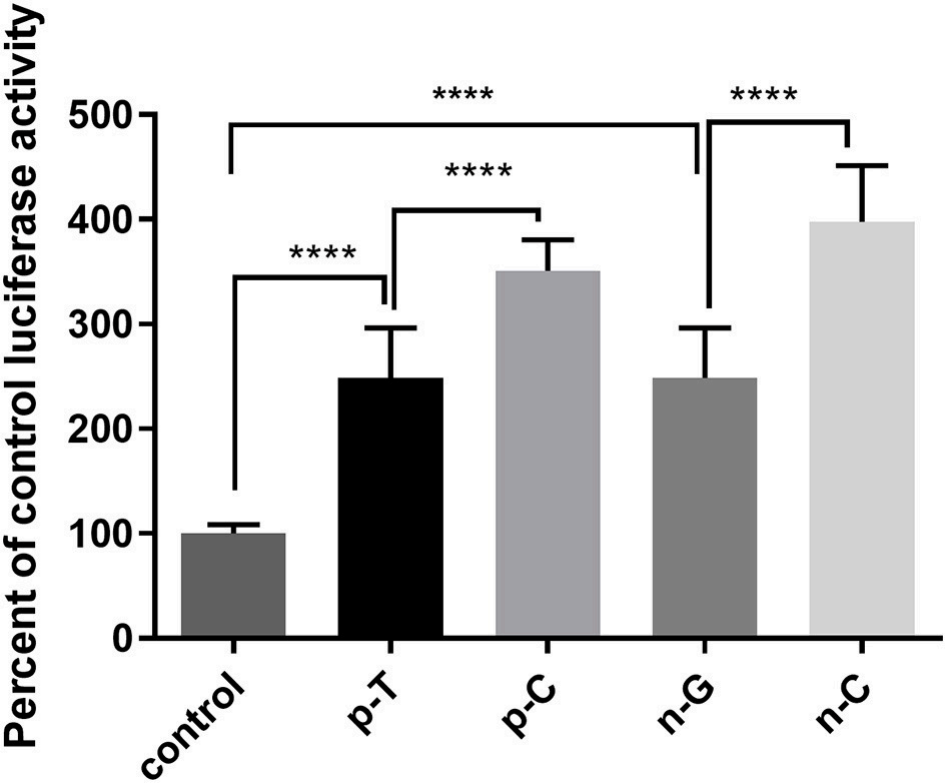
Luciferase assay of rs1024611 and GRCh38.p12chr17:.34252593 G>C. SH-SY5Y cells were transfected with p-C, p-T (rs1024611 SNPs), n-G, n-C (GRCh38 p12chr17:34252593), or pGL3-basic empty plasmid (as a negative control) and transcriptional activity was assessed using a luciferase assay. The control had significantly less transcriptional activity compared to wildtype and mutant alleles (*p* <0.0001). The C alleles for both SNPs had significantly greater transcriptional activity compared to their wildtype controls (^****^p < 0.0001).

The C allele of rs1024611 and C allele of GRCh38.p12chr17: 34252593 showed an association with PD risk. Since these two SNPs are located in the promoter region of CCL2, a dual-luciferase reporter gene assay was conducted to test whether the rs1024611 C allele and GRCh38.p12chr17: 34252593 C allele alter CCL2 promoter transcriptional activity. As shown in Figure 2, a significant higher induction of luciferase activity was observed in the presence of p-C or n-C, which represent the PD-risk allele of the promoter region of CCL2. Thus, cells with the rs1024611 C allele or GRCh38.p12chr17: 34252593 C allele might upregulate CCL2 expression.

### Meta-Analysis for rs1024611, rs4073, and rs2280788

**Figure 3:**
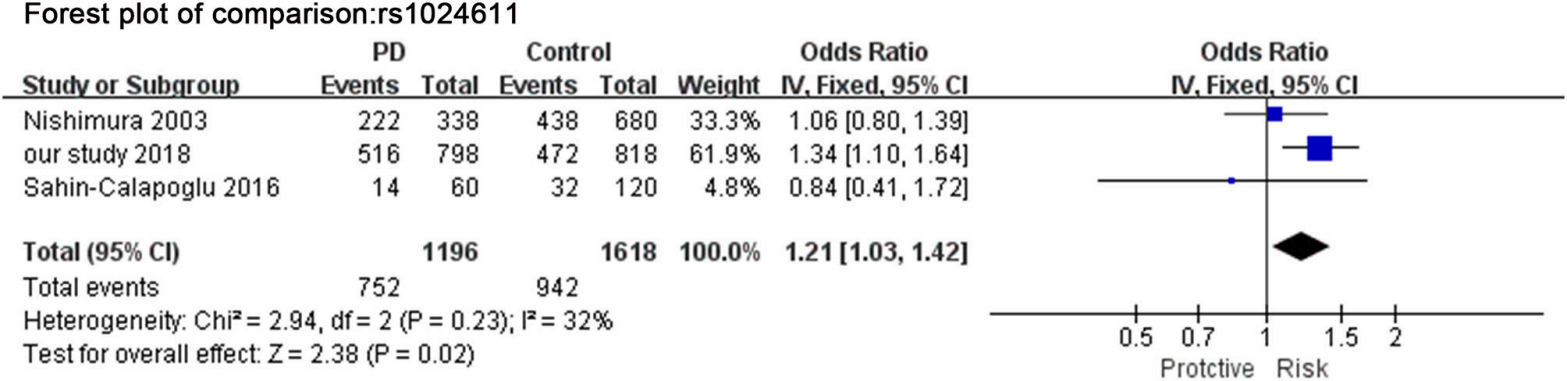
Meta-analysis for the association of rs1024611 with PD. Forest plot for the association between *rs1024611* polymorphism and genetic susceptibility to PD (CC vs. Total). Squares boxes indicate the odds ratios and the size of the box is proportional to the weight of the study. Dashed vertical lines represent the null value (OR = 1.0). Horizontal lines represent the 95% confidence intervals.

A total of 3 studies (including our study) in Figure 3 assessed the relationship between SNP rs1024611 and PD. The Q-statistic did not indicate significant heterogeneity between allele C and allele T (*I*^2^ = 32%). There was significant difference between allele C and allele T (OR = 1.21, 95%CI [1.03,1.42], *p* = 0.020).

## Discussion

PD is a multifactor disease caused by a combination of environmental and genetic factors. Polymorphisms in certain genes might contribute to neuro-inflammation pathogenesis and increase the susceptibility to sporadic PD (21). SNPs related to the expression level of inflammatory factors are also widely involved in PD (12, 13). To the best of our knowledge, this is the first case control study in a Chinese Han population to follow up the recent finding on SNPs in CCL2. This case control study included 411 PD patients and 422 controls, focus on neuro-inflammation-related gene, CCL2.

Intriguingly, two significant alterations in CCL2, rs1024611, and GRCh38.p12chr17:34252593 G>C were identified in this study. The C allele of rs1024611 of the CCL2 gene was significantly higher in PD patients (64.66%) than that in controls (57.50%), suggesting its association with an increased risk of PD (*p* = 0.024). Based on meta-analysis, allele C was still significant relative to PD risk (*p* = 0.010) in a cohort of 784 PD patients and 998 controls. Besides, rs1024611 has been reported as a pathogenic allele in other neuroinflammatory CNS diseases, including Alzheimer's disease and ischemic stroke (22, 23). In addition, the alteration GRCh38.p12chr17:34252593 G>C, which has not been reported previously, was identified in 7 PD patients (1.70%), while none of the controls carried this alteration. There is no linkage disequilibrium between allele C of rs1024611 and allele C of GRCh38.p12chr17:34252593 (data not show).

Furthermore, in subgroup analysis, risk allele C of rs1024611 was found to be significantly increased in male PD patients but not in female PD patients. This means this risk allele appears to be more relevant to male PD patients but not females and male individuals who carry this allele are with greater susceptibility to PD.

Five of seven PD patients carrying allele C of GRCh38.p12chr17:34252593 were diagnosed with AR form of PD (*p* = 0.005), and this subtype PD usually shows a faster progression and more cognitive decline (24). Thus, allele C of GRCh38.p12chr17:34252593 might be helpful to predict AR subtype in clinical diagnosis. However, the larger-scale cohort study is needed to confirm this relationship between this SNP and AR subtype of PD.

Because there are 55 patients and 170 controls have hypertension medical history, 13 patients and 45 controls have diabetes medical history in our cohort. To see if these two chronic disease effects our conclusion, we analysis the relationship between these SNPs and PD again after removing the subjects with HTN or D2M. Without HTN subjects, allele C of rs1024611 as well as allele C of GRCh38.p12chr17:34252593 G>C was still significantly higher in PD patients than those in controls (*p* = 0.003 and *p* = 0.043, respectively). Similarly, when D2M cases were excluded, both allele C of rs1024611 and allele C of GRCh38.p12chr17:34252593 G>C were found to be significantly higher in PD patients than those in controls (*p* = 0.003 or *p* = 0.002). Therefore, rs1024611 allele C and GRCh38.p12chr17:34252593 allele C are still risk factor of PD after these two effects are excluded.

CCL2, a chemokine that participates in neuro-inflammation is expressed by multiple cells in the central nervous system (CNS) such as astrocytes, microglia, and neurons. Parillaud et al. found that moderate CCL2 (CCL2) over-expression led to increased neurotoxicity in MPTP treated mice, likely due to increased CCR2^+^ monocyte infiltration in the CNS (25). CCL2 was also found to promote apoptosis and secretion of TNF-α and IL-1β in neuroblastoma SH-SY5Y cells and inhibit cell viability, while the knockdown of CCL2 exerted the opposite effects (26). Down-regulating CCL2 *in vivo* was found to markedly relieve MPTP-induced movement disorder and spatial memory deficits and to play neuroprotective and anti-inflammatory roles in MPTP-induced PD mice (10). All these results indicate that the level of CCL2 is positively correlated to the risk of PD.

According to the transcription factor binding site predicted on CONSITE, rs1024611 and GRCh38.p12chr17:.34252593 G>C are closely adjacent to the two NFκB binding sites which are essential for the cytokine induced CCL2 expression (data not shown). We tested the transcriptional activity of CCL2 promoter region using a luciferase reporter assay and found that the C allele variants of rs1024611 and GRCh38.p12chr17:34252593 showed higher transcriptional activity than the original alleles (T and G, respectively). Even though these two loci are not directly located in the region of the transcription factor binding sites, Farley et al. found that the syntax pattern, which means the sequence surrounding the transcription binding site, including the length and composition of bases, is crucial to the expression of genes. Transcription factors might prefer certain spatial arrangements and conformations of the binding site, or certain bases simply have a higher affinity to the transcription factor (27). Combining these results with our experimental results, we propose that the risk alleles of these two SNPs lead to higher CCL2 transcriptional activity than their original alleles. Patients carrying these SNPs might have higher levels of CCL2 and an increased risk of PD.

In summary, this study is the first case-control study in a Chinese Han population to explore the connection between specific SNPs in the promoter region of CCL2 and PD. However, because of our limited statistical power, larger-scaled, and multi-center case control studies are needed to provide stronger evidence, and functional experiments are needed to confirm the role of our newly identified SNP.

## Author Contributions

JD and SC: project design. SC, YW, and LH: samples collection. RS and XZ: genetic analysis. RS, SL, and ZZ: statistical analysis. JD and RS: manuscript writing. All authors have approved the final article to be submitted.

### Conflict of Interest Statement

The authors declare that the research was conducted in the absence of any commercial or financial relationships that could be construed as a potential conflict of interest.
