# Corrigendum: Association of Two Polymorphisms in CCL2 With Parkinson's Disease: A Case-Control Study

**DOI:** 10.3389/fneur.2019.00300

**Published:** 2019-04-02

**Authors:** Ruinan Shen, Suzhen Lin, Lu He, Xue Zhu, Zhekun Zhou, Shengdi Chen, Ying Wang, Jianqing Ding

**Affiliations:** ^1^Institute of Neurology, Ruijin Hospital, Shanghai Jiao Tong University School of Medicine, Shanghai, China; ^2^Shanghai Jiaotong University School of Medicine, Shanghai, China

**Keywords:** CCL2, polymorphism, Parkinson's disease, Chinese population, risk factor

In the original article, there was a mistake in Figure 3 as published. In the enrolled study group Sahin-Calapoglu 2016, the event number (allele C) of Parkinson's Disease (PD) and the control was wrongly included in the calculation. Where it reads “46” in PD and “88” in the control, it should be “14” in PD and “32” in the control, therefore the final heterogeneity between allele C and allele T is *I*^2^ = 32%. Despite the error, there was still a significant difference between allele C and allele T (OR = 1.21, 95%CI [1.03,1.42], *p* = 0.020). The corrected Figure 3 appears below.

**Figure 3:**
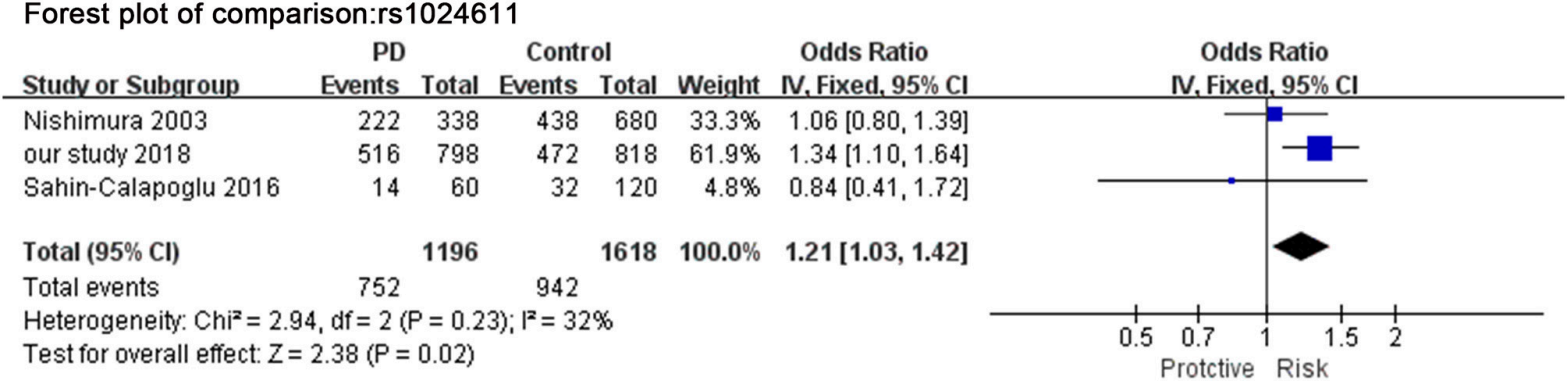
Meta-analysis for the association of rs1024611 with PD. Forest plot for the association between *rs1024611* polymorphism and genetic susceptibility to PD (allele C vs. Total). Squares boxes indicate the odds ratios and the size of the box is proportional to the weight of the study. Dashed vertical lines represent the null value (OR = 1.0). Horizontal lines represent the 95% confidence intervals.

Due to the error in Figure 3 mentioned above, a correction has been made to the **Results**, **Meta-Analysis for rs1024611, rs4073, and rs2280788**.

“A total of 3 studies (including our study) in Figure 3 assessed the relationship between SNP rs1024611 and PD. The Q-statistic did not indicate significant heterogeneity between allele C and allele T (*I*^2^ = 32%). There was significant difference between allele C and allele T (OR = 1.21, 95%CI [1.03,1.42], *p* = 0.020).”

Additionally, in the original article, there was an error in figure legend of Figure 3 as published. Instead of “CC vs. Total,” it should be allele “C vs. Total.” The corrected Figure 3 legend appears as below.

The authors apologize for these errors and state that they do not change the scientific conclusions of the article in any way. The original article has been updated.

